# A Lab-in-a-Fiber optofluidic device using droplet microfluidics and laser-induced fluorescence for virus detection

**DOI:** 10.1038/s41598-022-07306-0

**Published:** 2022-03-03

**Authors:** Helen E. Parker, Sanghamitra Sengupta, Achar V. Harish, Ruben R. G. Soares, Haakan N. Joensson, Walter Margulis, Aman Russom, Fredrik Laurell

**Affiliations:** 1 Laser Physics Group, Department of Applied Physics, Royal Institute of Technology (KTH), 100 44 Stockholm, Sweden; 2 Scottish Universities Physics Alliance (SUPA), Institute of Photonics and Quantum Sciences, Heriot-Watt University, Edinburgh, EH14 4AS UK; 3 AMOLF, Science Park 104, 1098 XG Amsterdam, The Netherlands; 4 Science for Life Laboratory, Division of Nanobiotechnology, Department of Protein Science, Royal Institute of Technology (KTH), 171 65 Solna, Sweden; 5 Research Institutes of Sweden (RISE), 164 19 Stockholm, Sweden; 6 AIMES - Center for the Advancement of Integrated Medical and Engineering Sciences at Karolinska Institutet and KTH Royal Institute of Technology, Stockholm, Sweden

**Keywords:** Microfluidics, Fibre optics and optical communications, Optical sensors

## Abstract

Microfluidics has emerged rapidly over the past 20 years and has been investigated for a variety of applications from life sciences to environmental monitoring. Although continuous-flow microfluidics is ubiquitous, segmented-flow or droplet microfluidics offers several attractive features. Droplets can be independently manipulated and analyzed with very high throughput. Typically, microfluidics is carried out within planar networks of microchannels, namely, microfluidic chips. We propose that fibers offer an interesting alternative format with key advantages for enhanced optical coupling. Herein, we demonstrate the generation of monodisperse droplets within a uniaxial optofluidic Lab-in-a-Fiber scheme. We combine droplet microfluidics with laser-induced fluorescence (LIF) detection achieved through the development of an optical side-coupling fiber, which we term a periscope fiber. This arrangement provides stable and compact alignment. Laser-induced fluorescence offers high sensitivity and low detection limits with a rapid response time making it an attractive detection method for in situ real-time measurements. We use the well-established fluorophore, fluorescein, to characterize the Lab-in-a-Fiber device and determine the generation of $\sim$ 0.9 nL droplets. We present characterization data of a range of fluorescein concentrations, establishing a limit of detection (LOD) of 10 nM fluorescein. Finally, we show that the device operates within a realistic and relevant fluorescence regime by detecting reverse-transcription loop-mediated isothermal amplification (RT-LAMP) products in the context of COVID-19 diagnostics. The device represents a step towards the development of a point-of-care droplet digital RT-LAMP platform.

## Introduction

Microfluidic technologies offer powerful solutions for biologists to manipulate and analyze biological samples in miniaturized systems with a high degree of control. Microfluidic chips feature sub-millimeter channel systems supplied with liquid from connected reservoirs. Chip design and parallelization facilitates rapid and multimodal processing and interrogation of molecular or cellular species in a single device and has, together with automated mass-production, enabled commercial uses in diagnostics, environmental analysis^1^ and bioscience^2–4^. Droplet microfluidics is a microfluidic technology which utilizes discrete aqueous droplets, separated by an immiscible oil, and stabilized by surfactants, as microscale sample containers. Monodisperse droplets can be generated and interrogated within the microfluidic system. Digital detection and analysis of single cells or molecules in nano to pico-liter sample packets can be performed at ultra high-throughput (> 1000 droplets s$^{-1}$). Droplets have been used in vitro to encapsulate a variety of targets such as bacteria^5^ and cells^6^, and to perform highly sensitive virus detection^7,8^.

Droplet partitioning compartmentalizes a single sample into thousands of droplets which are environmentally isolated from one another. On one hand, partitioning a sample at the moment it is collected allows for high resolution temporal and spatial analysis of the sampled material in situ, in vivo, as demonstrated previously for chemical monitoring^9–11^. On the other hand, partitioning a single dilute sample into many droplets, each containing either a single target molecule or lacking target molecules enables digital detection of an amplified molecular species, significantly reducing the demands on optical detectors in *e.g.* droplet digital PCR^12^ and digital droplet LAMP^13^. The detection and quantification of droplet-encapsulated material may involve the use of optics integrated into the chip. So called optofluidic devices offer a way to synergistically combine key microfluidics sample processing capabilities with optical functionality, opening up promising avenues for bio- and chemical-sensing^14^. Established optical techniques such as fluorescence-activated droplet sorting^15,16^, optical microscopy^17,18^, or laser-induced fluorescence (LIF)^19–21^, can be incorporated into microfluidic chips.

Integrating microfluidic capacity directly into optical fibers and capillaries offers an alternative emerging route to benchtop optofluidics. Optical fibers possess a set of attributes which include flexibility, chemical and bio-inertness, easy light coupling, and a high aspect ratio. Moreover, their widespread use across numerous fields, from telecommunications to sensing^22,23^ and imaging in medicine^24–27^ have brought about high tolerance, and standardized production in kilometer lengths which can be assembled into low-cost optofluidic devices with precise channel dimensions using equipment developed for telecommunications production. In previous work, we demonstrated several all-fiber optofluidic devices for biomedical applications fabricated from an array of optical fibers and capillaries. We demonstrated devices for the detection, trapping, and collection of fluorescent microparticles^28,29^ and demonstrated the potential of these techniques by developing a low-cost all-fiber flow cytometer^30^. Most recently, we have demonstrated particle separation and counting in an uninterrupted flow through a fiber device^31^. Furthermore, structured hollow core fibers allow for the guiding of light directly in the fluid media, which has been used for Raman sensing^32^ and for the tracking of diffusion of nanoscale objects^33,34^ including virus particles^35^. Structured optical waveguides, such as light cages, have been developed for absorption spectroscopy with possible applications in bioanalytics^36^. These seamlessly integrated Lab-in-a-Fiber optofluidic devices indicate potential for extending the use of Lab-in-a-Fiber technology to molecular diagnostics.

Starting in late 2019, the SARS-CoV-2, or COVID19, pandemic represented a major global health challenge and continues to have far reaching societal and economic consequences. The pandemic has highlighted the need for globally deployable, fast and scalable biomolecular testing^37^ for high as well as low-income countries. The tests developed for ongoing SARS-CoV-2 infection include rapid antigen tests, which detect the presence of key viral proteins in nasopharyngeal swab specimens, and nucleic acid tests which amplify and detect viral genetic material in nasal, throat or mouth swabs. Of these, nucleic acid amplification tests are expected to provide higher sensitivity and potentially higher specificity than rapid antigen tests^38^.

In this work, motivated by fortifying and broadening the range of test formats for sample-to-answer medical diagnostics, we have developed a droplet microfluidic Lab-in-a-Fiber device to carry out two key bioassay functions for a rapid nucleic acid amplification test based on digital LAMP. These two steps are: droplet encapsulation of sample material, and in situ detection of optical signatures through LIF. Features of our Lab-in-a-Fiber device are: droplets are generated in a negative pressure regime (a requisite for in situ sampling); LIF excitation and detection is carried out on board within a novel compact fiber arrangement; and operation of the device is carried out via hardware connected at only one end, simplifying the experimental layout and freeing up the length of the Lab-in-a-Fiber device. We describe the fabrication of this Lab-in-a-Fiber device and its characterization using a dilution series of fluorescein solutions from 100 $\upmu$M to 10 nM. As a proof-of-concept demonstration, we present results from a pre-amplified RT-LAMP-based nucleic acid amplification SARS-CoV-2 assay. Thus, we demonstrate that the integrated Lab-in-a-Fiber device operates within a diagnostically relevant fluorescence detection regime for such nucleic acid amplification tests.

## Methods

This section describes: the fabrication of the Lab-in-a-Fiber device; the optical detection setup; and the experimental methods used to characterize the complete setup using fluorescein and a pre-amplified RT-LAMP assay.

### Fabrication of the Lab-in-a-Fiber device

**Figure 1:**
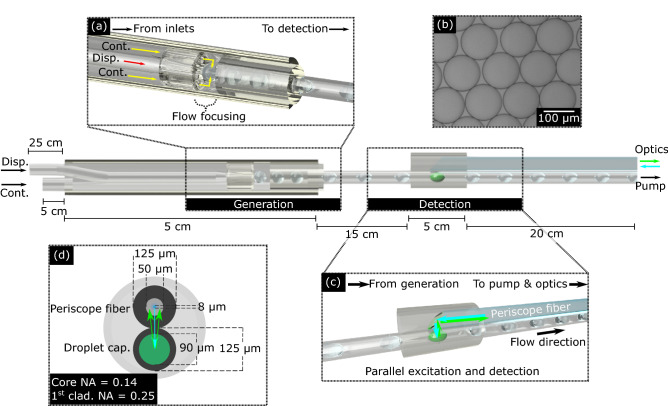
Overview of the Lab-in-a-Fiber device. The device consists of two modules. (**a**) Dispersed (disp.) phase droplets generated via flow focusing. The central hole of a five-hole capillary was fed with the dispersed phase and the exterior four holes were fed with the continuous (cont.) phase oil. A secondary capillary placed in close proximity to the output of the five-hole capillary forced the oil into the dispersed phase path, facilitating droplet generation via flow focusing. (**b**) Widefield microscopy image of droplets produced from 1 $\upmu$M fluorescein. They were measured to be approximately 120 $\upmu$m in diameter. (**c**) Fluorescence detection of droplets. Delivery of excitation light and collection of fluorescence emission was achieved on-axis using a periscope fiber (a double-clad fiber (DCF) cleaved at an angle of $45^\circ$ and coated with aluminum). (**d**) Cross-sectional view of the periscope fiber secured in parallel to the droplet capillary. The 8 $\upmu$m core of the periscope fiber was used to couple continuous wave (CW) laser light to the droplets beneath, as indicated by the blue arrow. The induced fluorescence was collected through the 50 $\upmu$m $1{\mathrm{st}}$ cladding region, as indicated by the green arrows. Note that the length of the Lab-in-a-Fiber device is not shown to scale, but indicative lengths of each section are given.

The Lab-in-a-Fiber device was made from a catalogue of fibers and capillaries drawn in-house and assembled using a Vytran Automated Glass Processor Workstation (GPX3000, Thorlabs). The device schematic is shown in Fig. 1 and comprises two modules with the following roles: droplet generation via flow-focusing; and optical detection. A detailed description of the fabrication process of each module is given below. The Lab-in-a-Fiber device operates with proximal-end light coupling and application of negative pressure via a syringe pump, allowing for distal-end sample loading.

#### Droplet generation module

**Figure 2:**
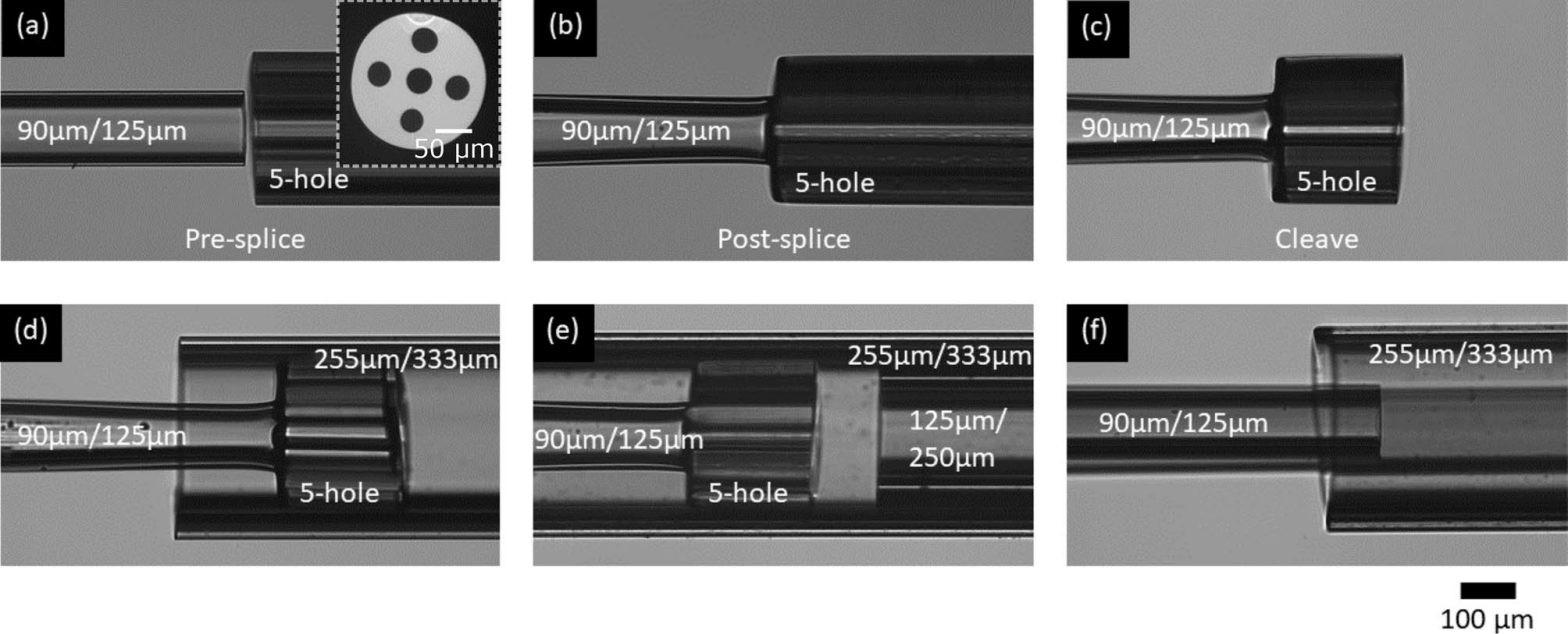
Droplet generation module. Fabrication steps of the droplet generation module of our fiber device shown in Fig. 1(a). (**a**) A 90 $\upmu$m/125 $\upmu$m capillary was aligned to the central hole of a five-hole capillary (end face shown inset), where the five holes were 30 $\upmu$m in diameter each. (**b**) The capillaries were fusion spliced ensuring that the four outer holes of the five-hole capillary remained open and the 90 $\upmu$m/125 $\upmu$m capillary did not collapse. (**c**) The five-hole capillary was cleaved to a length of approximately 200 $\upmu$m. (**d**) The five-hole capillary section was inserted into the left side of a 255 $\upmu$m/333 $\upmu$m housing capillary. (**e**) A 125 $\upmu$m/250 $\upmu$m capillary was inserted into the right side of the housing capillary and was brought to a distance of approximately 100 $\upmu$m from the exit holes of the five-hole capillary. (**f**) A second 90 $\upmu$m/125 $\upmu$m capillary was inserted into the left side of the housing capillary.

A compound capillary was made by first aligning a 90 $\upmu$m/125 $\upmu$m inner diameter/outer diameter (ID/OD) capillary to the central hole of a five-hole capillary (Fig. 2a). Each of the holes of the five-hole capillary had an ID of 30 $\upmu$m and were arranged like the dots on the five-side of six-faced dice (Fig. 2a (inset)). The 90 $\upmu$m/125 $\upmu$m ID/OD capillary was then fusion spliced to the central hole (Fig. 2b). The splice process slightly collapsed the 90 $\upmu$m/125 $\upmu$m ID/OD capillary so that the four outer holes were not blocked but the 90 $\upmu$m/125 $\upmu$m ID/OD capillary remained open. The five-hole capillary was then cleaved to a length of 200 $\upmu$m from the splice position (Fig. 2c). This compound capillary section formed the droplet generation element and was inserted from the left side into a 5 cm long section of a 255 $\upmu$m/333 $\upmu$m ID/OD housing capillary (Fig. 2d). A 5 cm long section of 125 $\upmu$m/255 $\upmu$m ID/OD capillary was inserted from the right side of the housing capillary and its end face was brought to a distance of 100 $\upmu$m from the cleaved end of the five-hole capillary (Fig. 2e). This distance influences the produced droplet size^39,40^ and was determined empirically. Following this, a second 90 $\upmu$m/125 $\upmu$m ID/OD capillary was inserted a short way into the left hand entrance of the housing capillary to provide oil supply for the outer four holes of the five-hole capillary (Fig. 2f). Both ends of the housing capillary were sealed with UV-cured glue (4-20418-GEL, Dymax) (not shown), leaving the short length of the 125 $\upmu$m/255 $\upmu$m ID/OD capillary open. Thus the 90 $\upmu$m/125 $\upmu$m ID/OD capillary spliced to the central hole of the five-hole capillary served as the dispersed/aqueous phase inlet and the second 90 $\upmu$m/125 $\upmu$m ID/OD capillary served as the continuous/oil phase inlet. This is illustrated in Fig. 1a.

We used basic hydrodynamic resistance calculations to select suitable lengths of each inlet such that the droplets were generated with sufficient separation from one another. This was 1:3.5 (continuous phase inlet length:dispersed phase inlet length)), which, in our device, was 7 cm and 25 cm, respectively. The entire arrangement resulted in monodisperse sets of droplets of 120 $\upmu$m in diameter (Fig. 1b). A 40 cm long piece of 90 $\upmu$m/125 $\upmu$m ID/OD capillary was inserted and secured with UV-cured glue in the open end of the 125 $\upmu$m/255 $\upmu$m ID/OD capillary. The droplet detection module was then built onto this length of capillary. As a final step in the fabrication of the droplet generation module, a siliconizing reagent, Sigmacote^®^ (SL2, Sigma-Aldrich), was flushed steadily through the device for 45 min. This ensured that the interior walls of the flow channels were appropriately hydrophobic and fluorosilicic to prevent unwanted interaction between them and the disperse aqueous liquid.

#### Droplet detection module

**Figure 3:**
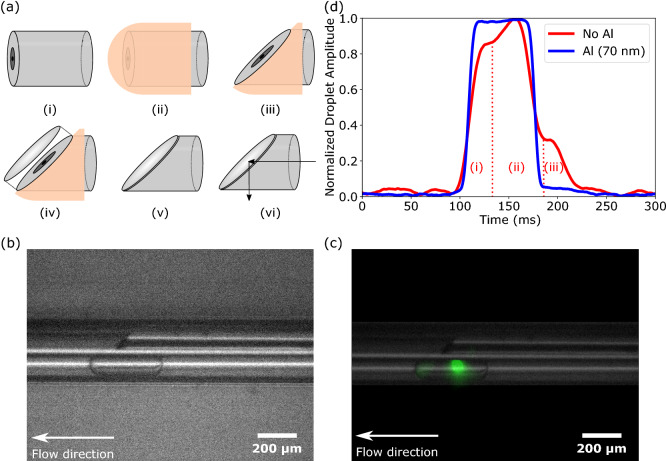
Droplet detection module. (**a**) Step-by-step process of fabricating the periscope fiber. (i) The double-clad fiber (DCF) was flat-cleaved. (ii) A vacuum-safe paint was used to mask the tip. (iii) The DCF was cut in a dicing machine at an angle of $45^\circ$ normal to the fiber axis. (iv) The exposed cut surface was coated with aluminum. (v) The mask was removed with acetone and sonication. (vi) Light coupled to the proximal end of the fiber exits perpendicularly to the propagation axis at the tip. (**b**) White light microscope image of droplet detection module with periscope fiber affixed above the droplet capillary (CW laser off), see SI Video 1. (**c**) Fluorescence microscope image of same field of view as (**b**) (CW laser on) with transparent overlay of the device for clarity. Green fluorescence can be seen localized in the droplet under the periscope fiber, see SI Video 2. (**d**) Normalized 1 $\upmu$M fluorescein droplet profiles using a Lab-in-a-Fiber device built using a periscope fiber without aluminum layer (red line) and with aluminum layer (blue line).

Optical interrogation was performed within the Lab-in-a-Fiber device itself using a periscope fiber as illustrated in Fig. 1c. A double-clad fiber (DCF), drawn in-house, consisting of an 8 $\upmu$m core (numerical aperture (NA) of 0.14), a light-guiding 50 $\upmu$m $1{\mathrm{st}}$ cladding region (NA of 0.25), and a 125 $\upmu$m $2{\mathrm{nd}}$ cladding region was flat-cleaved (Fig. 3a(i)) and the tip was masked using vacuum-safe paint (Fig. 3a(ii)). The masked tip was then cut at an angle of $45^\circ$ normal to the fiber propagation axis using a dicing saw (DAD-2H/6M, Disco) (Fig. 3a(iii)). The DCF was then loaded into a thermal evaporator (Auto 306/FL400, Edwards) and the tip was coated with aluminum until a 70 nm layer of aluminum built up on the angled cut surface (Fig. 3a(iv)). The DCF was then immersed in acetone and sonicated for 20 min to remove the vacuum-safe paint, leaving behind a mirrored angled tip (Fig. 3a(v)). The periscope fiber was then secured within a housing capillary in parallel to and rotationally aligned with the droplet flow capillary instead of perpendicularly to it. Light propagated through the side of the fiber tip, as shown by the arrows in Figs. 1d and 3a(vi), analogous to a periscope. Thus, the fluorescence of each droplet could be measured independently while maintaining a uniaxial design, see Fig. 3b,c and SI Videos 1 and 2. From the 0.14 NA of the 8 $\upmu$m core, we calculated that the illumination region had an approximate width of 30 $\upmu$m within the droplet capillary. The 0.25 NA of the 50 $\upmu$m 1${\mathrm{st}}$ cladding region collects fluorescence from a region of approximately 80 $\upmu$m. Small reflections within the droplet capillary were observed, see left of droplet in Fig. 3c, however these were outside of the collection NA.

Even without aluminium deposition on the angled cut surface of the periscope fiber, sideways propagation of light from the tip was observed. However, the result was two local regions of light: a primary illumination region at $90^\circ$ to the propagation axis, and a secondary illumination region at an oblique angle which extended ahead of the fiber tip. This resulted in droplet peaks consisting of three sub-peaks as the droplets moved through the two illumination regions, shown in Fig. 3d (red line), representing: the leading end of the droplet coincident with the primary illumination region (Fig. 3d(i)); the body of the droplet coincident with both the primary and the secondary illumination region (Fig. 3d(ii)); and the trailing end of the droplet coincident with the secondary illumination region (Fig. 3d(iii)). Aluminum deposition ensured that light was better reflected to the primary region and this resulted in peaks closer to a top hat shape, see Fig. 3d (blue line).

### Optical setup

**Figure 4:**
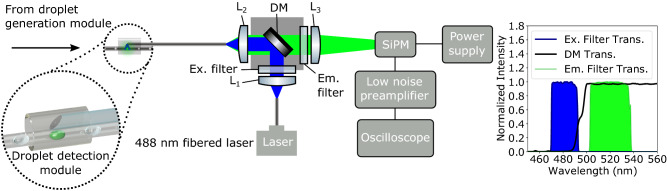
Optics and hardware for operating the Lab-in-a-Fiber device. Excitation from a 488 nm fiber-coupled laser was collimated using a fiber port ($L_{1}$). The light passed through an excitation filter (Ex. filter) and was reflected from a dichroic mirror (DM) to a second fiber port ($L_{2}$) which was used to couple the light into the Lab-in-a-Fiber device. Collected fluorescence passed through the dichroic mirror (DM) and an emission filter (Em. filter). The normalized transmission spectra for the filter set are shown on the right. An achromatic condenser lens ($L_{3}$) loosely focused the light onto a silicon photomultiplier (SiPM). The SiPM signal was coupled to an oscilloscope via a low noise pre-amplifier which was used as a 1 kHz low pass filter.

The optical setup was built from off-the-shelf components and is shown in Fig. 4. Excitation light from a 20 mW 488 nm single-mode CW fiber laser (LP488-SF20, Thorlabs) was collimated via an achromatic fiber port ($L_1$) (PAF2-A4A, Thorlabs). The light was passed through an excitation filter (Ex. filter) (MDF-GFP2 Excitation, Thorlabs) and reflected from a dichroic mirror (DM) (MDF-GFP2 Dichroic, Thorlabs). A second achromatic fiber port ($L_2$) (PAF2-A4A, Thorlabs) coupled the light into the central core of the periscope fiber. Since the mode field diameter of the fibered laser was 3 $\upmu$m we slightly defocused ($L_2$) in order to not underfill the 8 $\upmu$m core of the periscope fiber while also taking care not to guide excitation light through the $1{\mathrm{st}}$ cladding region. This was achieved by pre-aligning a dummy DCF fiber in the system and checking central core coupling using a camera at the distal end. Once good pre-alignment was achieved, the dummy DCF was replaced by the device. Avoiding cladding coupling minimized high order mode illumination of the droplets which would have resulted in an unstable incident intensity profile across the droplet cross-section. Microscope images showed a localized laser spot incident on the droplet itself. Thus, as droplets passed, their cross-sections were scanned along the capillary axis. Any misalignment of the laser light into the $1{\mathrm{st}}$ cladding region could be seen by a broadening of the laser spot incident on the droplet, coupled with an unstable fluorescence measurement, so could easily be identified and realigned if needed. Fluorescence induced in the droplets was coupled back through the periscope fiber via the core and the $1{\mathrm{st}}$ cladding region, which ensured maximized collection efficiency. The fluorescence was passed through the dichroic mirror (DM) (MDF-GFP2 Dichroic, Thorlabs) and filtered using an edge pass filter (Em. filter) (FELH0500, Thorlabs). An aspheric condenser lens ($L_3$) (ACL2520U-A, Thorlabs) was used to loosely focus the collected fluorescence onto a silicon photomultiplier (SiPM) (PM3325-WB, Ketek). The signal was first passed through a pre-amplifier (SR560, Stanford Research Systems), which we used as a 1 kHz low pass filter, and onto an oscilloscope (MSO54 5-BW-1000 Mixed Signal Oscilloscope, Tektronix) for readout.

### Experimental procedure

The full experimental procedure consisted of three steps: a continuous phase flushing step, a sample loading step, and a second continuous phase flushing step. For the first flushing step, both the dispersed phase inlet and the continuous phase inlet at the distal end of the Lab-in-a-Fiber device (left end of Fig. 1) were submerged in separate Eppendorf tubes each containing oil. At the proximal end (right end of Fig. 1) the droplet flow capillary was secured to a 2.5 mL syringe and loaded onto a syringe pump (AL-1000, World Precision Instruments). The pump was then set to a 12 $\upmu$L min$^{-1}$ withdrawal rate and was kept constant throughout the experiment. This rate was determined empirically to produce monodisperse droplets (of $\sim$ 0.9 nL from 1 $\upmu$M fluorescein solution) at a generation rate of 41 ± 0.5 Hz which was sustained for the entire experimental duration.

As a first step, the Lab-in-a-Fiber device was allowed to run for twenty minutes before sample loading, aspirating fluorinated oil through both inlets. For the sample loading step, the dispersed phase inlet was quickly lifted out of its oil Eppendorf tube and lowered into an Eppendorf tube containing the sample: in our case either fluorescein (for characterization experiments) or pre-amplified RT-LAMP SARS-CoV-2. The continuous phase inlet remained unmoved. The total sample volume was withdrawn before the final flushing step. For the final flushing step, the dispersed phase inlet was quickly lifted out of the sample Eppendorf tube and returned to its original oil Eppendorf tube and allowed to flush for twenty minutes. In the case of pre-amplified RT-LAMP SARS-CoV-2 experiments, we carried out the experiment both at room temperature and $65\,^\circ$C. The latter was achieved by securing the Lab-in-a-Fiber device to a homemade hot plate with a thermocouple and PID controller. This thermocouple provided live feedback of the temperature and the PID controller actively maintained $65\,^\circ$C. We measured the hot plate to have a stability of $65\,^\circ$C $\pm \,1\,^\circ$C throughout the experiment. We did not determine a minimal time for fluorinated oil flushing before or after sample loading.

### Data analysis

Analysis of droplet data was performed offline using relevant Python packages according to the following method: background fluoresence (which was determined to originate primarily from the periscope fiber itself) was captured during each experiment and was subtracted from the raw data. Then a Savitzky-Golay filter was applied to the raw data and local maxima were found using a find peaks function^41^. The mean and standard deviation of the peak maxima^42^ and the mean and standard deviation of peak widths (FWHM of droplet peaks) were calculated.

### Patent

A patent related to the fiber arrangement for illumination and detection was filed on 22nd March 2021 to the European Patent Office. The patent filing number is (EP21164119).

## Materials

### Fluorinated oil

A 2.5% wt/vol solution of 008 fluorosurfactant (RAN Biotechnologies) very-long-chain surfactant molecules were dissolved in NOVEC 7500 engineering oil (3M Technology) and used as our continuous phase throughout all experiments.

### Fluorescein

Fluorescein dye (Sigma-Aldrich) was used to prepare solutions of different concentrations. 0.66 mg of fluorescein dye was measured using a weighing balance (ABS-120-4N, KERN) and was dissolved in 20 mL of PBS buffer solution (pH 7.2) (Alfa Aesar) to prepare a 100 $\upmu$M stock solution. The solution was homogenized by ultrasonication for one minute using an ultrasonication bath (Selecta Ultrasonic). Using the serial dilution method six lower concentrations of fluorescein solutions (10 $\upmu$M, 1 $\upmu$M, 500 nM, 100 nM, 10 nM, 1 nM) were prepared from the 100 $\upmu$M stock solution. Each solution was homogenized using ultrasonication for one minute before dilution.

### RT-LAMP of in vitro synthesized RNA fragment of SARS-CoV-2

Amplification of RNA was performed similarly to that described in Soares et al.^43^. The RT-LAMP was performed in an Eppendorf nexus gradient Mastercycler with a total of 100 $\upmu$L per reaction mixture. The reaction mixture containing 10 $\upmu$L template RNA (103 copies $\upmu$L^−1^) or DI-water was prepared with final concentrations of 1x Isothermal amplification buffer II (New England Biolabs), 6 mM dNTPs (RP65, Blirt), 6 mM MgSO_4_ (New England Biolabs), 1x primer mixture (six primers described in detail below), 1x EvaGreen^®^ (Biotium, 20x solution in water), 3.2 U Bst 3.0 polymerase (New England Biolabs) and 40 U SuperScript IV reverse transcriptase (Thermo Fisher Scientific). The primer mixture at 10x concentration was prepared with 2 $\upmu$M forward (F) and backward (B) primers, 16 $\upmu$M forward inner (FIP) and backward inner (BIP) primers and 4 $\upmu$M forward loop (LF) and backward loop (LB) primers. The iLACO primer sequences, reported by Yu et al.^44^, were the following: F-5′- CCA CTA GAG GAG CTA CTG TA-3′; B- 5′-TGA CAA GCT ACA ACA CGT-3′; FIP- 5′-AGG TGA GGG TTT TCT ACA TCA CTA TAT TGG AAC AAG CAA ATT CTA TGG-3′; BIP- 5′-ATG GGT TGG GAT TAT CCT AAA TGT GTG CGA GCA AGA ACA AGT G-3′; LF- 5′-CAG TTT TTA ACA TGT TGT GCC AAC C-3′; LB- 5′-TAG AGC CAT GCC TAA CAT GCT-3′. All primers were synthesized by Thermo Fisher Scientific. The RNA template (226 bp) matching the sequence of the ORF1ab of SARS-CoV-2 (MT883505.1, 15118-15343) used was produced by in vitro transcription and stored at $-80\,^\circ$C in DI-water at a concentration of 5 ng $\upmu$L^−1^.

## Results

### Characterization using fluorescein

**Figure 5:**
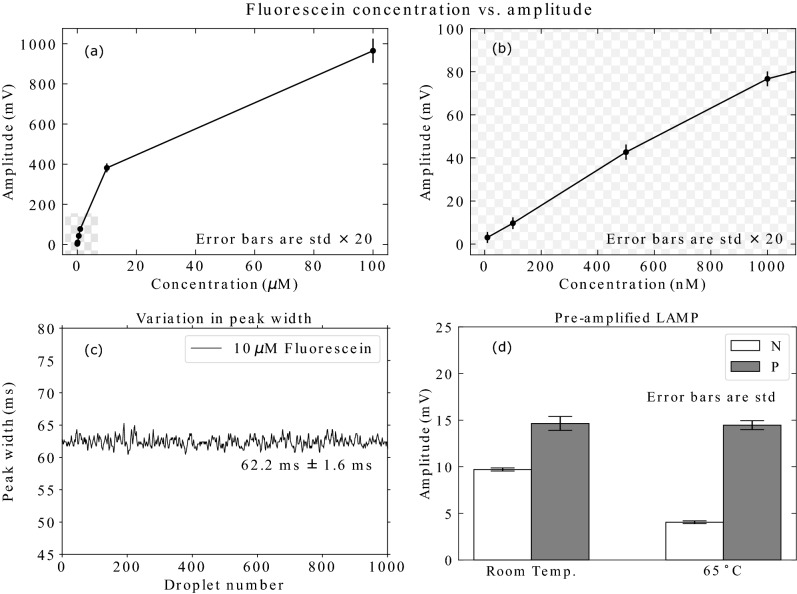
Characterization and results using an RT-LAMP assay. (**a**) Characterization of the Lab-in-a-Fiber device using fluorescein as a surrogate fluorophore. Each point represents 20,000 droplets and error bars represent standard deviation × 20 for visibility. Quenching of the fluorophore could be seen at high concentrations, as expected. (**b**) Zoomed in region from checkerboard area in (**a**), which shows the linear fluorescence regime at lower concentrations, before quenching effects are observed. (**c**) Representative measurement of droplet peak widths across 1000 consecutive droplets, demonstrating stable droplet velocities. (**d**) Results for pre-amplified SARS-CoV-2 RT-LAMP samples. White fill shows sample negative for RNA (N). Gray fill shows sample positive for RNA (P). The experiment was conducted at (left) room temperature and (right) at $65\,^\circ$C. Negative and positive were distinguishable from one another even at room temperature, but suppression of background fluorescence in the negative sample at $65\,^\circ$C increased the resolution.

In order to characterize the detection of droplets in the Lab-in-a-Fiber device, we selected fluorescein as an appropriate fluorophore surrogate for EvaGreen^®^. From our experience of the fluorescence of RT-LAMP products and fluorescein, whereby negative SARS-CoV-2 RNA RT-LAMP, positive SARS-CoV-2 RNA RT-LAMP, and 500 nM fluorescein displayed RFUs of 6600, 8700, and 7800, respectively, we determined that the most appropriate set of fluorescein dilutions for our characterization should be: 100 $\upmu$M, 10 $\upmu$M, 1 $\upmu$M, 500 nM, 100 nM, 10 nM, 1 nM. These dilutions were run through the Lab-in-a-Fiber device and data were collected for sets of approximately 20,000 droplets per dilution. The amplitude and standard deviation were calculated for each of the droplet sets for each concentration, see Fig. 5a. From our characterization, we determined that it was possible to distinguish between each droplet set and we established a limit of detection (LOD) of 10 nM fluorescein (Fig. 5b). Our LOD was defined according to 3.29 $\times$ the standard deviation of the background signal, in our case fluorinated oil. It has been reported that droplet velocity can result in significant variation in fluorescence intensity of fluorescein droplets, particularly at slow velocities ($\le 2.5\,{\mathrm{cm}}\, {\mathrm{s}}^{-1}$)^45^. Therefore, as a post-acquisition quality check, peak widths were calculated throughout all experiments, see Fig. 5c. Peak widths provide a representation of droplet size and/or speed. These measurements ensured we could attribute observed differences in droplet fluorescence intensity to fluorophore concentration and not changes in droplet velocity in this slow droplet regime.

### Detection of in vitro synthesized RNA fragment of SARS-CoV-2 via RT-LAMP

To demonstrate that our Lab-in-a-Fiber device was operational within a relevant biological regime, pre-amplified positive and negative SARS-CoV-2 RNA RT-LAMP samples were tested. A clear difference was observed in the average fluorescence value of the negative (9.7 mV ± 0.2) and positive (14.6 mV ± 0.7) RT-LAMP samples at room temperature, see Fig. 5d (left), with a signal-to-noise ratio (SNR), here defined as the ratio of the fluorescence value of positive droplets to that of negative droplets, of 1.5. When the detection of the same sample was done at $65\,^\circ$C, an increased difference between the average amplitude of negative (4.0 mV ± 0.2) and positive (14.5 mV ± 0.5) RT-LAMP samples was observed, see Fig. 5d (right), with an SNR of 3.6. At room temperature, primer dimer generation is more significant due to lower hybridization stringency, so the observed increase in population resolution is due to a decreased background fluorescence in the negative sample at $65\,^\circ$C.

## Discussion

The aim of this work was to demonstrate that well-established microfluidics techniques could be translated into an all-fiber optofluidic arrangement. Hence, we focused on developing two fundamental capabilities that could apply to a plethora of potential use cases: (i) monodisperse droplet generation and (ii) sensitive LIF detection. These capabilities were further developed with certain criteria in mind: (i) that the design remains uniaxial and compact, and (ii) that the device operates under negative pressure. We achieved droplet generation using a flow-focusing approach and detection using a periscope fiber without compromising on device footprint. The diameter of the Lab-in-a-Fiber device was no greater than 333 $\upmu$m at any point along its length (at the generation and detection modules), with the rest of the device being 125 $\upmu$m in diameter. The sample inlet capillary was 25 cm in length in 125 $\upmu$m in diameter, which makes it flexible and easy to maneuver by hand for simple sample loading. Our device exhibits the conventional benefits of optical fibers and capillaries, namely, biological and chemical inertness, ready integration with conventional optics, and the potential to be developed towards in vivo use cases.

With the capabilities of fibers well demonstrated across the fields of telecommunications and biomedicine, it is exciting to consider the impact that Lab-in-a-Fiber technology could have in the field of optofluidics, which has so far been mostly explored within Lab-on-a-Chip architectures. Although Lab-in-a-Fiber research is in its nascent stage, one could imagine multiplexed functional fiber devices that incorporate, for example, microfluidics, sensing, and imaging to address a range of diagnostic challenges. In order to fully realize the potential of Lab-in-a-Fiber optofluidics, certain challenges, which we discuss below, will need to be addressed.

Thermal drawing techniques have led to the mass manufacturability of fibers and capillaries. This has already been exploited for biomedical device development. For example, imaging fibers can be drawn using relatively inexpensive materials^46,47^, enabling fiber-based endoscopy. It may be possible to address the bottleneck in our device fabrication process, namely, manual assembly of the constituent fibers and capillaries, with more sophisticated manufacturing techniques. For example, photolithography and etching could be used to make connective parts to facilitate the assembly process, ensuring tighter device-to-device repeatability while keeping down cost.

We determined a LOD of 10 nM fluorescein of our Lab-in-a-Fiber device. The main limiting factor of our LOD was the collection efficiency of our periscope fiber. The NA of the 1${\mathrm{st}}$ cladding region of the DCF was 0.25 and this resulted in a collection efficiency of $<\,5$% of the total fluorescence emission from the droplet. Greater collection efficiency could be achieved through the use of a DCF with a larger light-guiding cladding region, or with higher NA.

Capillary flow channels with a high aspect ratio, i.e., long and narrow, enable simple and easy sample loading but introduce a significant resistance which limits throughput. As described in the introduction, a key possibility of microfluidics is high-throughput analysis, with droplet generation possible at rapid rates (> kHz). In the results presented, our Lab-in-a-Fiber device produced droplets at a rate of 41 ± 0.5 Hz. Increasing the droplet rate could be achieved by shortening the device and increasing the inner diameter of the capillaries while still maintaining ease-of-use.

Although outside the scope of this work, it is also relevant to discuss how onboard amplification of the nucleic acid material could be achieved. The amplification would need to occur after droplet generation, either through a continuous flow isothermal temperature zone at $65\,^\circ$C or stationary in a large diameter capillary that provides sufficient volume to house and hold the sample plus continuous phase, in our case 10 $\upmu$L and 30 $\upmu$L, respectively. This capillary could be readily incorporated into our pre-existing design and then be heated from an external thermal source. Due to the wider research interests of our group, we chose RT-LAMP^43^ as an appropriate first demonstration of LIF detection.

## Conclusion

In this work we describe the development of a novel Lab-in-a-Fiber device and its potential application for droplet microfluidics. The fiber device is constructed from a variety of capillaries and fibers which are widely available, and we demonstrate that the device is functional for both the generation of monodisperse droplets ($\sim$ 0.9 nL) and their optical detection via laser-induced fluorescence. The Lab-in-a-Fiber device generates droplets using a uniaxial capillary flow-focusing arrangement through the application of negative pressure. Optical interrogation is performed on board through a double-clad fiber with an angled mirrored tip, which operates like a periscope to allow light coupling perpendicular to the fiber axis, keeping the device compact. The lowest detectable concentration that was tested was 10 nM fluorescein which is on par with many traditional Lab-on-a-Chip microfluidic platforms. Beyond our current work, we believe that further development could involve incorporating a modular temperature control unit and incubation chamber for onboard nucleic acid amplification.

## Supplementary Information

Supplementary Information 1.

Supplementary Information 2.

Supplementary Information 3.
