# Author Correction: A Lab-in-a-Fiber optofluidic device using droplet microfluidics and laser-induced fluorescence for virus detection

**DOI:** 10.1038/s41598-022-09240-7

**Published:** 2022-03-24

**Authors:** Helen E. Parker, Sanghamitra Sengupta, Achar V. Harish, Ruben R. G. Soares, Haakan N. Joensson, Walter Margulis, Aman Russom, Fredrik Laurell

**Affiliations:** 1 Laser Physics Group, Department of Applied Physics, Royal Institute of Technology (KTH), 100 44 Stockholm, Sweden; 2 Scottish Universities Physics Alliance (SUPA), Institute of Photonics and Quantum Sciences, Heriot-Watt University, Edinburgh, EH14 4AS UK; 3 AMOLF, Science Park 104, 1098 XG Amsterdam, The Netherlands; 4 Science for Life Laboratory, Division of Nanobiotechnology, Department of Protein Science, Royal Institute of Technology (KTH), 171 65 Solna, Sweden; 5 Research Institutes of Sweden (RISE), 164 19 Stockholm, Sweden; 6 AIMES - Center for the Advancement of Integrated Medical and Engineering Sciences at Karolinska Institutet and KTH Royal Institute of Technology, Stockholm, Sweden

Correction to: *Scientific Reports* https://doi.org/10.1038/s41598-022-07306-0, published online 03 March 2022

The original version of this Article contained an error in the spelling of the author Ruben R. G. Soares, which was incorrectly given as Ruben G. Soares.

This error has now been corrected in the original Article.

