# Supplementary material for: A Lab-in-a-Fiber optofluidic device using droplet microfluidics and laser-induced fluorescence for virus detection
Source: Sci Rep. 2022 Mar 3;12:3539. doi: 10.1038/s41598-022-07306-0 (PMC8894408; doi:10.1038/s41598-022-07306-0)
Supplement: Supplementary file 3 — Supplementary Information 3. [file 41598_2022_7306_MOESM3_ESM.pdf]

Supplementary Information:

SI Video 1 title: White light microscopy of detection module arrangement

SI Video 1 caption:

White light illuminated video showing the detection module embedded inside the Lab-in-a-Fiber device. Monodisperse droplets pass through the field of view within a 90 µm capillary. The periscope fiber (45 ° angle cut double-clad fiber) is secured in parallel to the droplet path by a housing capillary, facilitating excitation and collection of fluorescence from the droplets. Laser source is off.

SI Video 2 title: Fluorescence microscopy of detection module arrangement

SI Video 2 caption

Video showing the detection module embedded inside the Lab-in-a-Fiber device under a fluorescence microscope. Monodisperse 1 µM fluorescien droplets pass through the field of view within a 90 µm capillary. The periscope fiber (45 ° angle cut double-clad fiber) is secured in parallel to the droplet path by a housing capillary, facilitating excitation and collection of fluorescence from the droplets. A 488 nm laser coupled to the proximal end of the periscope fiber excites green fluorescence in the droplets, which is then collected in an epi-fluorescence arrangement through the periscope fiber.
